# A tea bud segmentation, detection and picking point localization based on the MDY7-3PTB model

**DOI:** 10.3389/fpls.2023.1199473

**Published:** 2023-09-28

**Authors:** Fenyun Zhang, Hongwei Sun, Shuang Xie, Chunwang Dong, You Li, Yiting Xu, Zhengwei Zhang, Fengnong Chen

**Affiliations:** ^1^ School of Automation, Hangzhou Dianzi University, Hangzhou, China; ^2^ Tea Research Institute, Shandong Academy of Agricultural Sciences, Jinan, China

**Keywords:** tea bud picking point, multi-attention mechanism, deep learning, DeepLabv3+, YOLOv7, focal loss

## Abstract

**Introduction:**

The identification and localization of tea picking points is a prerequisite for achieving automatic picking of famous tea. However, due to the similarity in color between tea buds and young leaves and old leaves, it is difficult for the human eye to accurately identify them.

**Methods:**

To address the problem of segmentation, detection, and localization of tea picking points in the complex environment of mechanical picking of famous tea, this paper proposes a new model called the MDY7-3PTB model, which combines the high-precision segmentation capability of DeepLabv3+ and the rapid detection capability of YOLOv7. This model achieves the process of segmentation first, followed by detection and finally localization of tea buds, resulting in accurate identification of the tea bud picking point. This model replaced the DeepLabv3+ feature extraction network with the more lightweight MobileNetV2 network to improve the model computation speed. In addition, multiple attention mechanisms (CBAM) were fused into the feature extraction and ASPP modules to further optimize model performance. Moreover, to address the problem of class imbalance in the dataset, the Focal Loss function was used to correct data imbalance and improve segmentation, detection, and positioning accuracy.

**Results and discussion:**

The MDY7-3PTB model achieved a mean intersection over union (mIoU) of 86.61%, a mean pixel accuracy (mPA) of 93.01%, and a mean recall (mRecall) of 91.78% on the tea bud segmentation dataset, which performed better than usual segmentation models such as PSPNet, Unet, and DeeplabV3+. In terms of tea bud picking point recognition and positioning, the model achieved a mean average precision (mAP) of 93.52%, a weighted average of precision and recall (F1 score) of 93.17%, a precision of 97.27%, and a recall of 89.41%. This model showed significant improvements in all aspects compared to existing mainstream YOLO series detection models, with strong versatility and robustness. This method eliminates the influence of the background and directly detects the tea bud picking points with almost no missed detections, providing accurate two-dimensional coordinates for the tea bud picking points, with a positioning precision of 96.41%. This provides a strong theoretical basis for future tea bud picking.

## 1 Introduction

Tea, made from the young shoots and leaves of the tea tree, is one of the most widely consumed beverages in the world (Yu and He, 2018). In 2018, global tea production reached 5.8 million tons, with China accounting for 45% of the total production and ranking first in the world. As tea production continues to increase annually, it poses a great challenge to the labor force. In order to address this trouble, researchers have developed relevant harvesting machines (Han et al., 2014; Du et al., 2018; Xia et al., 2022; Xu et al., 2022). However, manual harvesting remains the primary method for harvesting tea shoots, supplemented by machinery, and it requires a significant amount of time and labor. However, as labor costs increase, specialists become scarce, and tea producers demand higher quality, mechanized tea picking is becoming an inevitable trend for the sustainable development of the tea industry (Zhu et al., 2021). Therefore, using computer vision to quickly and accurately identify tea shoot picking in natural environments will become a key issue for intelligent tea picking.

In order to improve crop quality through mechanized picking, the first and fundamental task is to be able to identify shoots and then locate picking points in complex environments (Li et al., 2021). In fact, many experts and scholars have researched the detection and classification of crops and other plants (Zheng et al., 2017a; Zheng et al., 2017b). For example, Hai et al. (2014) established a tea shoot color distribution model to roughly separate tea shoot regions of interest (ROIs) from complex backgrounds, and then extracted local features around the top buds of tea leaves. These features were put into mean offset clustering to locate the tea shoot picking points. Lin et al. (2019) proposed a detection algorithm that utilizes color, depth, and shape information to detect spherical or cylindrical fruits on plants in natural environments, guiding harvesting robots to pick them automatically. Liu et al. (2019) used color features extracted from blocks to determine candidate regions and adopted the histogram of oriented gradient (HOG) to describe the shape of fruits, which achieved the average values of recall, precision, and F-1 reach 89.80%, 95.12%, and 92.38% respectively. Wang et al. (2019) utilized a natural statistical visual attention model to remove background saliency and combined it with a threshold segmentation algorithm to extract salient binary regions of apple images. Wu et al. (2019) presented a clustering and model segmentation based approach for detecting ripe peaches using RGB-D (red, green, blue, and depth space) cameras combined with color data and 3D contour features. As observed in the aforementioned study, the target objects are distinct from the background objects such as leaves in terms of color and shape, enabling easy recognition and extraction through color and shape features. However, the similarity in color and shape between tea buds and tea leaves, as well as the background of tea buds being tea leaves, makes it difficult for the human eye to distinguish between them. Consequently, traditional detection methods relying on color and shape are inadequate for accurately identifying tea buds within tea leaves.

In recent years, the development of artificial intelligence and 5G networks has had a significant impact on agriculture, as science and technology have continued to evolve. Furthermore, deep learning has opened up new possibilities for researchers in various fields, including target recognition. Wu et al. (2023) devised novel Inner Cascaded U-Net and Inner Cascaded U2-Net as improvements to plain cascaded U-Net for medical image segmentation, achieving better segmentation performance in terms of dice similarity coefficient and hausdorff distance as well as getting finer outline segmentation. Nie et al. (2022) proposed a method named SegNet that was developed and trained with different data groups. Quantitative metrics and clinical-based grading were used to evaluate differences between several groups of automatic contours. Yuan et al. (2022) proposed an enhanced network architecture based on PSPNet, referred to as Shifted Pool PSPNet, which integrates a module called Shifted Pyramid Pool instead of the original Pyramid Pool module to enable the utilization of entire local features for pixels located at the grid edges. Xie et al. (2023) proposed a population-based intelligent algorithm called Salp Swarm Algorithm for Feature Selection (SSAFS) for plant disease detection based on images. This algorithm aims to determine the optimal combination of handcrafted features and reduce the feature dimensionality to improve accuracy. Compared to the state-of-the-art algorithms, SSAFS demonstrates advantages in exploring the feature space and utilizing valuable features for plant disease image classification. Fu et al. (2021) proposed an improved DeepLabv3+ semantic segmentation algorithm for crack detection. The proposed method utilizes a densely connected spaceless pyramidal pooling module in the network structure, which enables the network to obtain more dense pixel sampling and improves the network’s ability to extract detailed features. Xie et al. (2022) proposed a new computational framework that combines deep embedding image clustering strategy, weighted distance measurement, and t-random neighbor embedding algorithm. The results indicate that the newly developed framework can identify plant diseases and uncover subtypes effectively, demonstrating excellent clustering performance. Yang et al. (2019) trained a model for detecting tea tree shoots using an improved “You Only Look Once” (YOLO) network. They achieved high precision results for the validation dataset. Xu et al. (2022) proposed a tea shoot recognition model based on a cascade network. First, the YOLOv3 network was used for the initial selection of tea shoot regions, and the resulting recognition outputs were then fed into DenseNet. Subsequently, the recognition results were further processed by the DenseNet-201 tea shoot classification network. The final recognition precision of tea shoots reached 95.71%, which provides a new approach to tea shoot recognition. Chen et al. (2022) proposed a tea-picking point location method based on YOLO-v3 algorithm, semantic segmentation algorithm, skeleton extraction, and minimum bounding rectangle. They designed an intelligent tea-picking machine based on personal computer and microcontroller cooperative control, which solved the problems of complex shadows and easy damage during the picking process. Jiang et al. (2022) proposed an improved attention mechanism YOLOv7 algorithm, named CBAM-YOLOv7, which adds three CBAM (Convolutional Block Attention Module) modules to the backbone network of YOLOv7 to enhance the network’s feature extraction capabilities. They also conducted comparison experiments using SE-YOLOv7 and ECA-YOLOv7. Yan et al. (2022) proposed the MR3P-TS model for tea shoot detection. The model calculates the area of multiple connected domains in the mask to determine the main part of the tea shoot. Then, it calculates the minimum bounding rectangle of the main part to determine the stem axis of the tea tree. Finally, the location coordinates of the tea shoot’s pickup point are obtained. Gui et al. (2023) proposed a lightweight tea leaf detection model based on an improved YOLOv5 architecture. The model incorporates the Ghost_conv module to reduce model size and includes the BAM module in the backbone network to suppress irrelevant information. The improved model achieved an average precision increase of 9.66% while reducing parameters by 22.71 M. Lu et al. (2023) proposed a method for segmentation and localization of tea buds. The method initially employs four semantic segmentation algorithms to process the images of tea buds. The position of the tea bud is determined by calculating the centroid and the centroid of the minimum bounding rectangle. This method achieves effective localization of tea buds. Qi et al. (2021) combined the “Maximum Between-Class Variance Method” (Otsu) with the traditional watershed algorithm to determine the threshold for image segmentation, thereby improving the accuracy of segmentation. They also improved the SE module to enhance the performance of deep learning networks and achieved outstanding accuracy on the tea bud dataset.

Therefore, the problem of segmenting, recognizing, and localizing objects with similar background colors and shapes, such as tea buds in complex environments, needs to be addressed, and precision needs to be further improved. In order to achieve precise identification and localization of tea buds, it is necessary to accurately obtain the two-dimensional coordinates of the image. After obtaining the two-dimensional coordinates, a specific coordinate system conversion is performed to ultimately obtain the three-dimensional spatial coordinates of the picking point. Therefore, accurate two-dimensional image coordinates play a crucial role in obtaining future three-dimensional spatial coordinates. To obtain accurate two-dimensional image coordinates, we establish an image dataset of tea buds in their natural growth state and propose a new model called the MDY7-3PTB model. The main contributions are as follows: (1) Focal Loss as a loss function to address data imbalance; (2) the improved DeepLabv3+ model with a fused CBAM attention mechanism for tea shoot segmentation in complex environments, removing interference from the background in tea shoot picking point detection; (3) accurate detection of tea shoot picking points based on YOLOv7;(4) the geometric center of the bounding box to achieve two-dimensional coordinate positioning of tea shoot picking points and restore their backgrounds.

## 2 Related work

### 2.1 DeepLabV3+ network segmentation: one of the top-performing semantic segmentation algorithms currently available.

**Figure 1:**
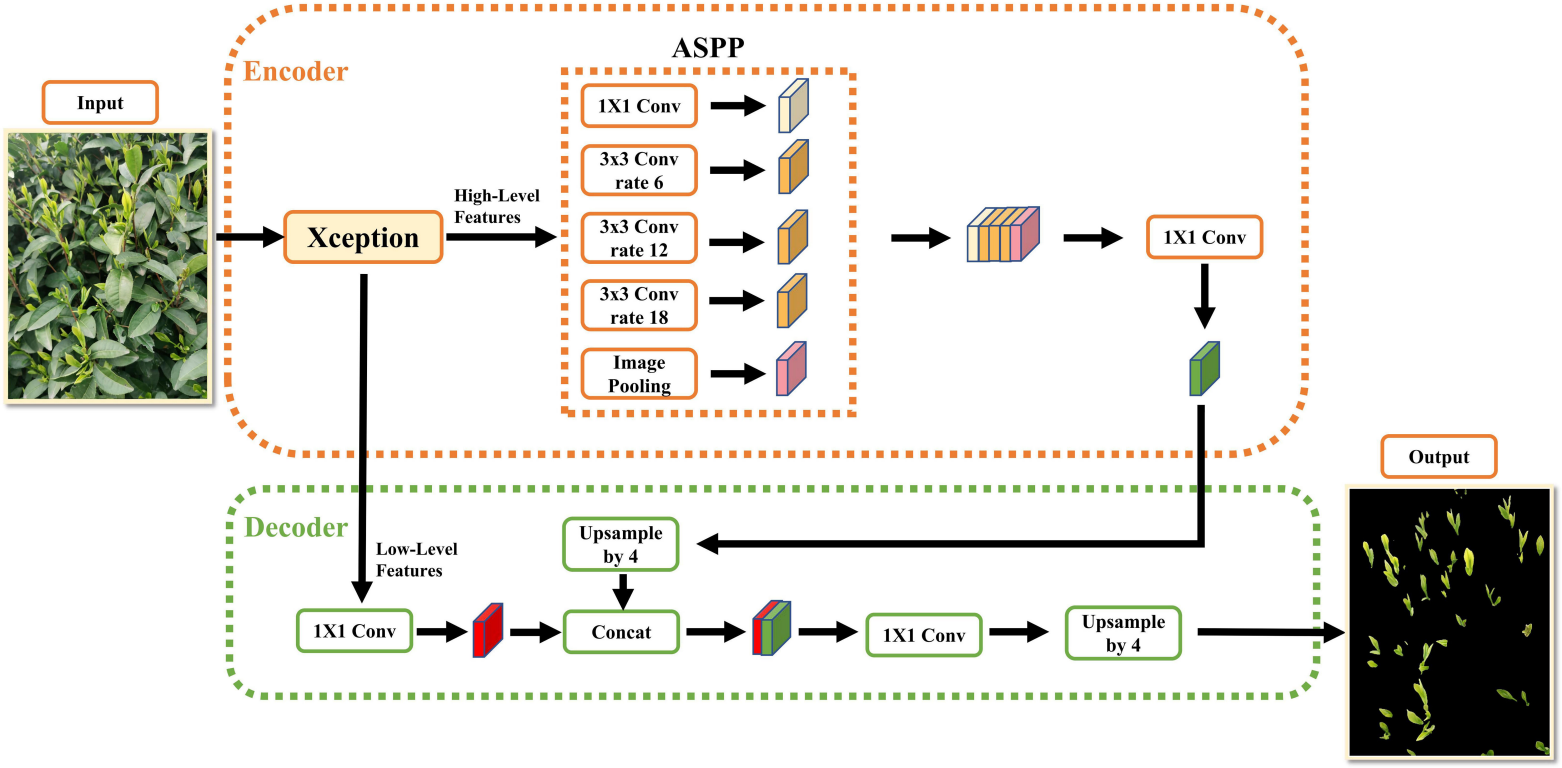
The original architecture of the DeepLabv3+ network.

The DeepLabV3+ network segmentation is formed by building the encoding and decoding structure upon the DeepLabv3 architecture with the addition of concise and effective decoders. The overall network structure is illustrated in **Figure 1**.

As can be seen from the figure, it is composed of an encoder network and a decoder network, which is similar to the structure of traditional semantic segmentation networks. At the encoder stage, the input image is subjected to initial feature extraction using the Xception backbone network that includes null convolutions to produce a low-order feature map that is 1/4 of the original image size and a high-order semantic feature map that is 1/16 of the original image size. The higher-order semantic feature maps are then fed into the ASPP module, which is composed of null convolutional layers with expansion rates of 6, 12, and 18 to capture the contextual global information of the feature maps. Finally, the ASPP module outputs the feature map for channel stitching. Since the feature map has a high feature channel dimension at this point, 1×1 convolution is used to downscale it to reduce network computation, and the downscaled feature map is passed into the decoder network.

In the decoding stage, the bilinear interpolation algorithm is used to upscale the feature map output from the encoder network by a factor of 4 to match the size of the low-level feature map. Then, the two feature maps are concatenated and fused for channel stitching. Finally, the fused feature maps are restored to the original image size using 3x3 convolution and 4 times up-sampling, and the final segmentation result is obtained by applying Softmax probability prediction (Zheng et al., 2022).

### 2.2 Attention mechanism: focusing on selective information

The attention mechanism is an algorithm that imitates the selective observation behavior of the human brain’s visual system. Its primary function is to assign larger weights to significant features to highlight their importance and smaller weights to irrelevant features to suppress the interference of irrelevant features during network training, thereby enhancing the learning ability of network models. In the tea shoot recognition task discussed in this paper, background noise can interfere with the recognition of tea shoots due to the relatively small number of shoot features and the lack of contrast with the background color. Thus, incorporating the attention mechanism into the tea shoot segmentation network can decrease the feature weight of background noise and enhance the representation of effective shoot features, thereby improving the network’s recognition and localization capability for tea shoots.

During the development of computer vision applications, numerous works on attention mechanisms have been proposed. One of these is the Convolutional Block Attention Module (CBAM), which was introduced by Woo et al. (2018) In 2018, this attention module combines channel attention and spatial attention to enable the network to highlight important features and suppress irrelevant features. The CBAM module differs from other attention modules in that it focuses on both channel and spatial dimensions, thereby achieving better efficiency.

The CBAM attention mechanism first employs the channel attention mechanism to enhance important channel features and suppress irrelevant ones. Then, the enhanced features are passed to the spatial attention mechanism to locate the region with the most informative features (in this case, the tea shoot region). Finally, the processed feature results are output. The formula can be expressed as follows:


(1) $${\text{F}}^{\prime }={\text{M}}_{c}(\text{F})⊗\text{F}$$

(2) $${\text{F}}^{\prime \prime }={\text{M}}_{s}({\text{F}}^{\prime })⊗{\text{F}}^{\prime }$$

In the formula, F∈ R^C × H × W^ represents the input feature map, where R represents the set of real numbers and C, H, and W represent the number of channels, height, and width of the feature map, respectively. M_c_∈R^C ×1×1^ represents the channel attention weights obtained by applying the channel attention mechanism, and M_s_(F’) ∈R^1× H × W^ represents the spatial attention weights obtained by applying the spatial attention mechanism to the transformed feature map F’.

In the channel attention module, the input feature map is compressed using a spatial dimension method, and the AvgPool and MaxPool methods are applied simultaneously to effectively calculate the weight attention assigned to the channel dimension. The formula can be expressed as follows:


(3) $${\text{M}}_{\text{c}}\left(\text{F}\right)\begin{array}{l} =\text{σ}(\text{MLP}(\text{AvgPool}(\text{F}))+\text{MLP}(\text{MaxPool}(\text{F})))\;\\ =\text{σ}({\text{W}}_{1}({\text{W}}_{0}({\text{F}}_{\text{avg}}^{\text{C}}))+{\text{W}}_{1}({\text{W}}_{0}({\text{F}}_{\text{max}}^{\text{C}}))) \end{array}$$

In the formula, σ represents the Sigmoid function. MLP refers to a multi-layer perceptron with a hidden layer whose operational weights are determined by W_0_ and W_1_, where W_0_ is activated by the ReLU (Rectified Linear Unit) function, ${\text{W}}_{0}\in R^{\frac{C}{r}\times C}$ , ${\text{W}}_{1}\in R^{C\times \frac{C}{r}}$ .

The spatial attention module focuses on the location of information in the image and is complementary to the previous module. Computationally, it first applies AvgPool and MaxPool operations on the channel axis and concatenates them into a meaningful feature descriptor. The two pooling operations aggregate channel information of a feature map to generate a two-dimensional map. Finally, a convolution operation is performed by the convolution layer to obtain the corresponding spatial feature map (Chen et al., 2022). The formula can be expressed as follows:


(4) $${\text{M}}_{\text{S}}\left(\text{F}\right)\begin{array}{l} =\text{σ}({\text{f}}^{7\times 7}(\left[\text{AvgPool}(\text{F});\text{MaxPool}(\text{F})\right]))\;\\ =\text{σ}({\text{f}}^{7\times 7}(\left[{\text{F}}_{\text{avg}}^{\text{S}};{\text{F}}_{\text{max}}^{\text{S}}\right])) \end{array}$$

In the formula,  σ  represents the Sigmoid function, $\int^{7\times 7}$ represents the 7 × 7 convolution kernel and ${\text{F}}_{\text{avg}}^{\text{S}},{\text{F}}_{\text{max}}^{\text{S}}\in {\text{R}}^{1\times \text{H}\times \text{W}}$ .

**Figure 2:**
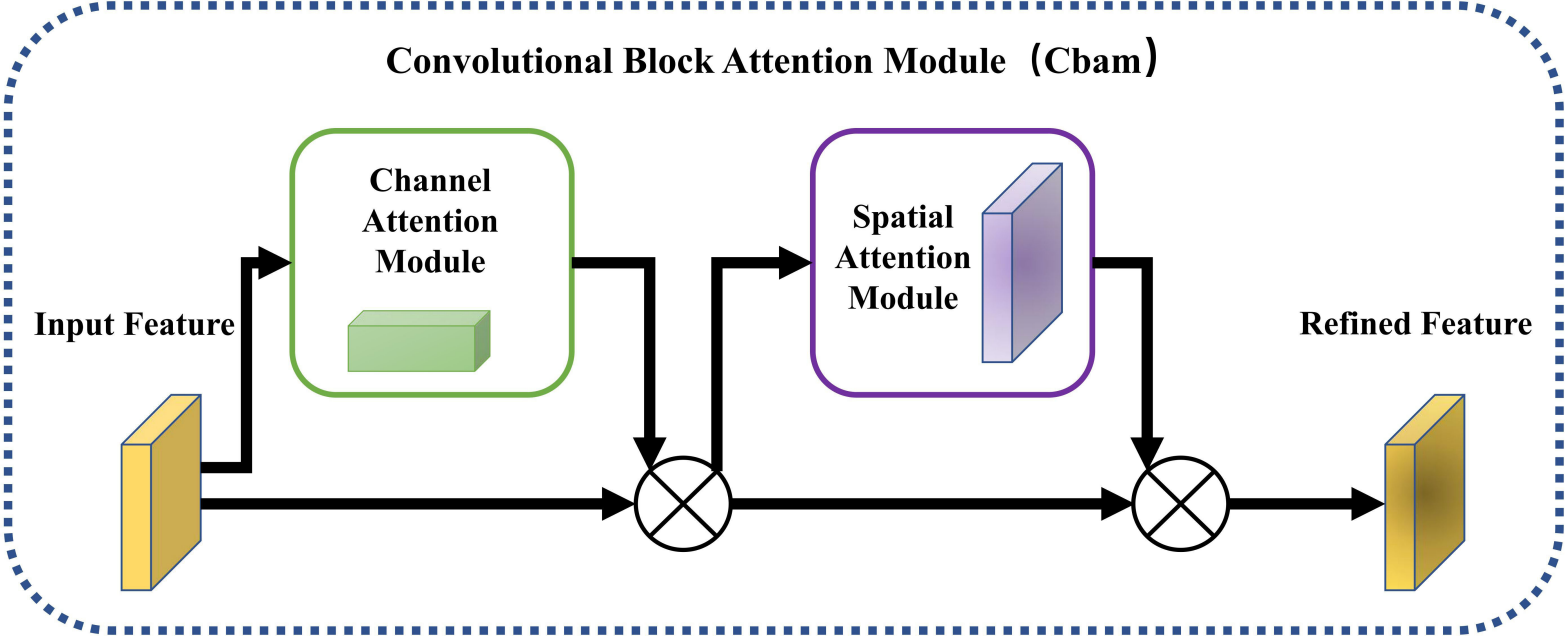
The schematic diagram of the CBAM attention mechanism.

To enhance the detection precision of the network, we incorporated the CBAM attention module into the backbone network structure, building upon its excellent performance. The resulting structure is illustrated in **Figure 2**.

### 2.3 YOLOv7: an excellent object detection model

**Figure 3:**
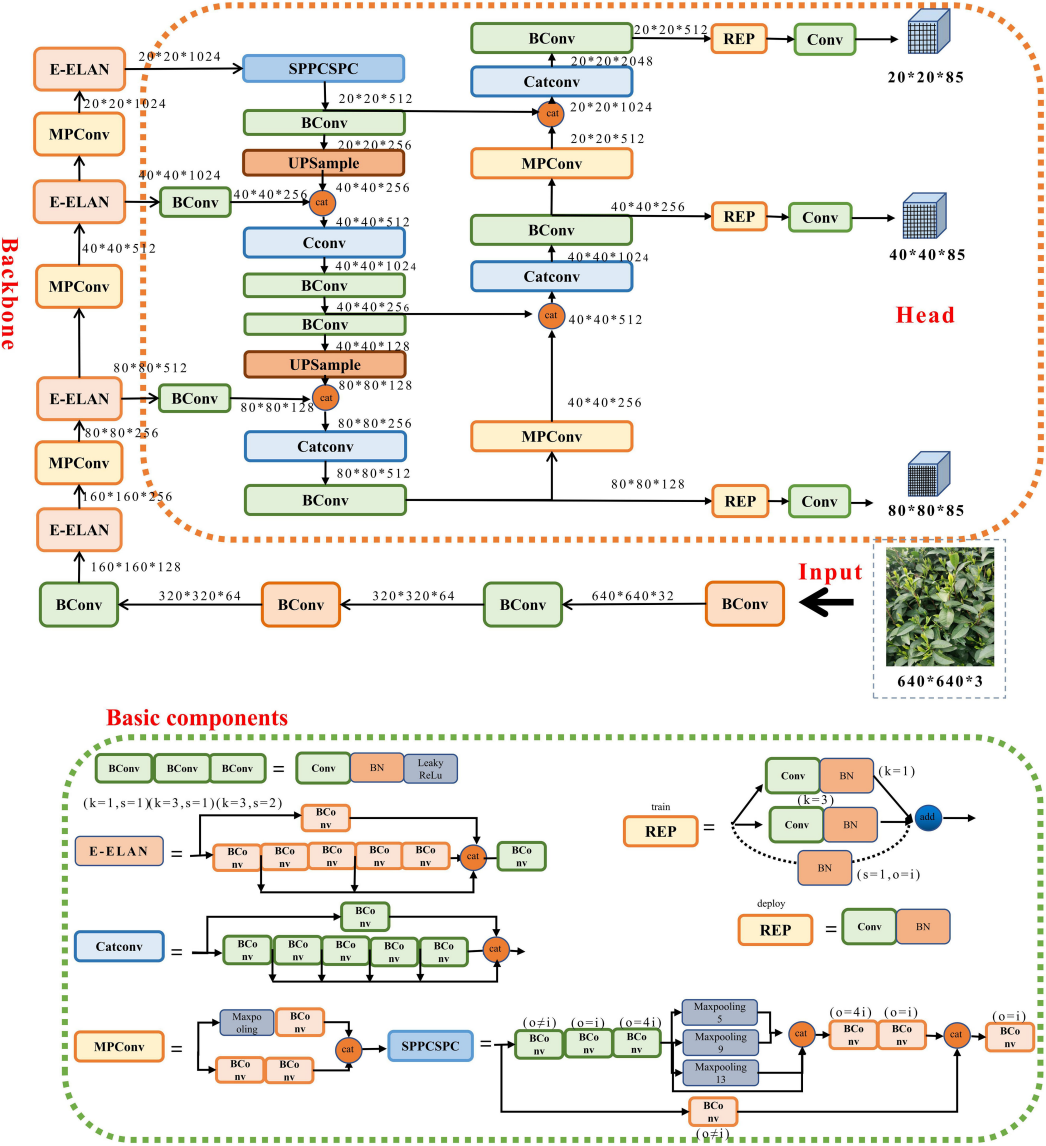
YOLOv7 network architecture.

In 2015, YOLOv1 (Redmon et al., 2016) was proposed, which introduced single-stage detection algorithms and effectively addressed the problem of slow inference speed in two-stage detection networks while maintaining good detection accuracy. YOLOv2 (Redmon and Farhadi, 2017) was an improvement over YOLOv1, with each convolutional layer being followed by a Batch Normalization layer, and dropout no longer being used. YOLOv3 (Redmon and Farhadi, 2018) was an improved version of the previous work, with its main feature being the introduction of the residual module darknet-53 and the FPN architecture, which predicted objects of three different scales and achieved multi-scale fusion. Based on the YOLOv3 version, YOLOv4 (Bochkovskiy et al., 2020) introduced PANet networks, mosaic data enhancement and CIoU loss function. Subsequently, YOLOv5 (Glenn, 2020) introduced various data enhancement methods, C3 modules, SPPF spatial pyramid pooling and Focal Loss loss function. In 2022, YOLOv7 (Wang et al., 2022) was introduced, which is currently one of the best detection models. It innovatively proposed the Extended-ELAN architecture, which can improve the network’s self-learning ability without breaking the original gradient path. Additionally, it adopts a model scale-based cascade method, which can generate models of corresponding scales for practical tasks to meet detection requirements. To more clearly express the internal composition of its network, the structure of each part of the YOLOv7 model is shown in **Figure 3**.

## 3 Materials and methods

### 3.1 Data acquisition

**Figure 4:**
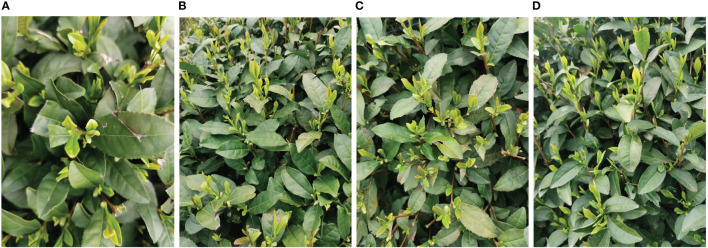
Images of tea buds were obtained under different conditions in complex environments. **(A)** close range, **(B)** long range, **(C)** top shot and. **(D)** side shot.

This paper focuses on the study of tea bud images in complex environments, and the tea bud samples used in this study were obtained from the Hangzhou Tea Research Institute in Zhejiang Province in 2022. The images were captured using the high-definition camera of a Xiaomi 10 mobile phone, with parameters shown in **Table 1**. In order to obtain more authentic and high-quality tea bud images in natural environments, the data was collected in mid-to-late March when the weather was clear and sunny outdoors. During the process of collecting tea bud image data, we changed the angle and distance of the camera multiple times to collect tea bud data from different directions and distances, and manually filtered out highly repetitive and blurred data. The collected tea bud images varied in pose, covering various distances, angles, and directions. Some of the collected tea bud images are shown in **Figure 4**.

**Table 1:** The camera parameters of Xiaomi 10 mobile phone.

Parameter	Value
Camera manufacturer	Xiaomi
Camera Model	Mi 10
Resolution	3000×4000
Aperture value	f/1.7
Exposure time	1/1001 sec
ISO speed	ISO-50
Exposure compensation	0 stops aperture
Focal length	7mm
Maximum aperture	1.51
Metering mode	Off-center average
Flash mode	No flash, forced
Image type	JPG

### 3.2 Data preprocessing

#### 3.2.1 Correction of category imbalance

Although this paper only focuses on the segmentation of tea buds into two categories (tea bud or background), the small size of the tea buds in the images and their small proportion in the overall image lead to a significant class imbalance, with a large proportion of the background class. This imbalance can result in inefficient training and negatively impact the model’s precision. Therefore, it is necessary to correct this class imbalance in the dataset. In this paper, we use the Focal Loss loss function to perform category imbalance correction. The formula can be expressed as follows:


(5) $$\begin{array}{c} \text{FL}\left({\text{P}}_{\text{t}}\right)=-{\text{α}}_{\text{t}}{\left(1-{\text{P}}_{\text{t}}\right)}^{\text{γ}}\text{log}\left({\text{P}}_{\text{t}}\right)\; \end{array}$$

(6) $${\text{P}}_{\text{t}}=\{\begin{array}{c} \text{p}\;\text{if}\;y=1\\ 1-\text{p}\;\text{otherwise} \end{array}\;$$

In the formula, P represents the detection result, y represents the true label, $\alpha_{t\;}$ represents the weighting factor, ${\left(1-P_{t}\right)}^{\gamma }$ represents the adjustment factor and γ ≥0 is the adjustable focusing parameter.

In this paper, we experimentally adjust the parameters $\;\alpha_{t}=0.5,\gamma =2$ .

#### 3.2.2 Image annotation

Due to the high density of tea bud picking points and the similarity in color between tea buds and tea leaves, it is challenging to annotate a large number of original images, leading to low prediction results and missed detections during model training and prediction. To overcome these issues, this study proposes segmenting all content other than tea buds as background, which makes the color of tea buds stand out and facilitates the image labeling, model training, and prediction. This approach improves overall efficiency and accuracy.

**Figure 5:**
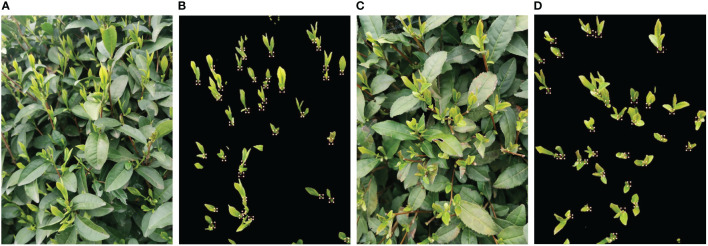
Examples of labeling results for tea bud picking points. **(A, B)** and **(C, D)** represents the original image and its annotation process.

In the detection phase, the segmented dataset is utilized to train the YOLOv7 model. Image annotation, being the fundamental component, significantly impacts the training and prediction of the model. Therefore, the annotation process is divided into four stages. The initial step involves manual labeling of partially segmented images. Subsequently, the partially labeled and all unlabeled images are uploaded to the EasyData platform for intelligent labeling in the second stage. In the third step, the images that are not fully labeled or unlabeled are identified and grouped with the manually labeled images. The fourth step involves filtering the labeled images to ensure completeness. Finally, the labeled files with annotations are saved in the VOC format. **Figure 5** displays the effect of labeled tea bud picking point images.

#### 3.2.3 Data augmentation

**Figure 6:**
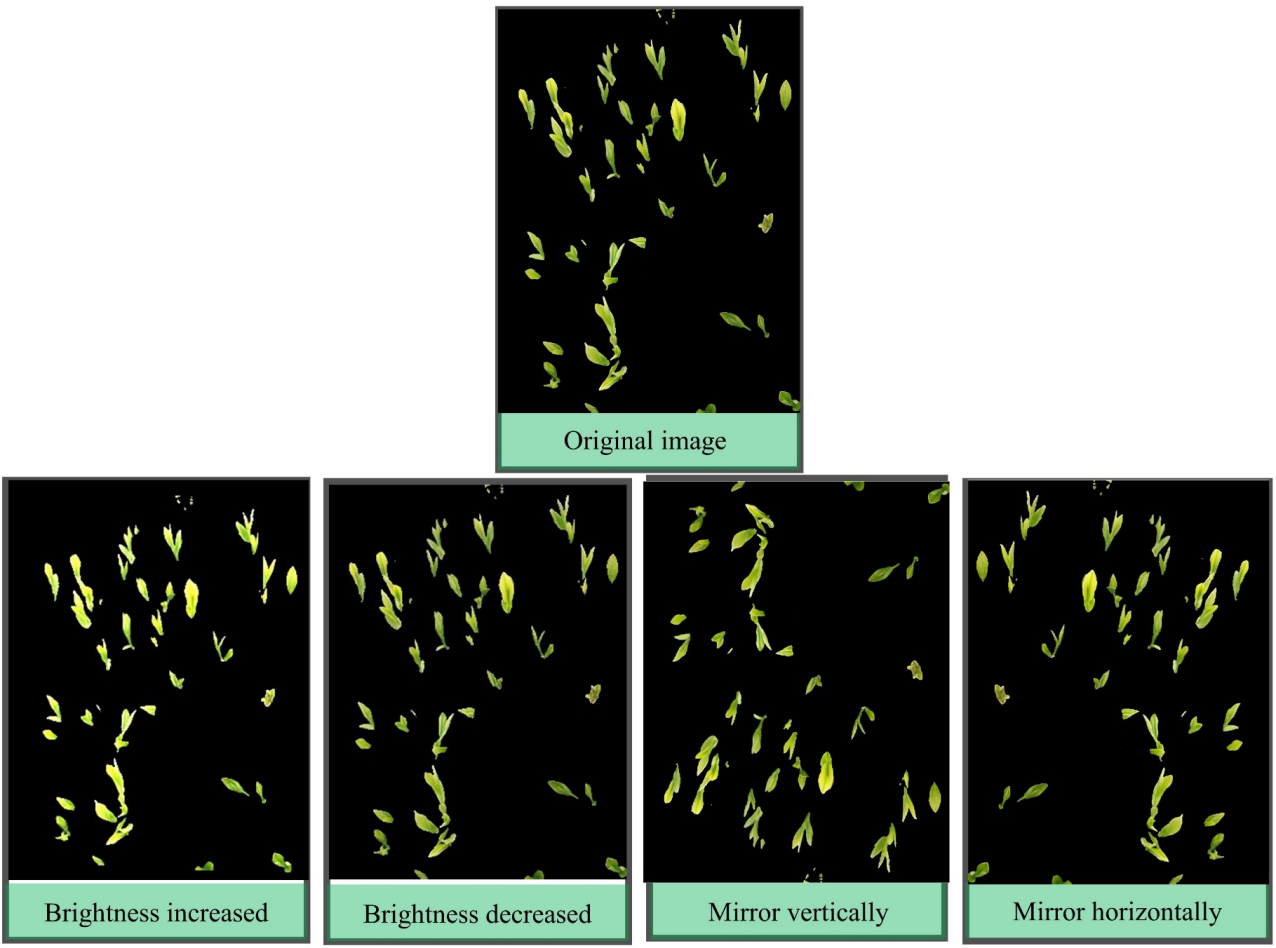
Results of data augmentation.

In deep learning object recognition networks, the amount of data often affects the final recognition performance, and too little data can easily lead to overfitting. For tea bud images, it is not enough to rely solely on downloading from the internet or collecting them personally, and the collection of tea bud images is also limited to around the Qingming Festival, which has temporal restrictions, and taking tea bud images in the field is also very time-consuming. Therefore, it is necessary to augment the tea bud image data. The specific operation of data augmentation is to expand the existing tea bud image by using relevant data augmentation methods before making the dataset, so as to achieve the effect of increasing the quantity of the dataset. Image brightness adjustment and flipping not only can expand the dataset but also improve the model’s robustness, accuracy, and generalization performance. Therefore, this paper used brightness increased, brightness decreased, horizontal flipping and vertical flipping for data augmentation (Xu et al., 2022) and the final dataset reached 2948 images. The image dataset was randomly divided into three groups to form the model training, validation and test datasets, with proportions of 70%, 20%, and 10%, respectively. These datasets will be used for model training and parameter optimization, and compared with the prediction results to evaluate the model’s object detection performance, as shown in **Figure 6**.

### 3.3 Experimental environment

In this paper, all experiments were conducted on the same computer, and specific information on the computer’s hardware and software configuration and model training environment is shown in **Table 2**.

**Table 2:** Computer hardware and software configuration and model training environment.

Environmental parameter	Value
CPU	AMD R9 4900H
GPU	GeForce 3060Ti
RAM	16GB
Video memory	6GB
Operating system	Windows10
Deep learning framework	PyTorch
Cudnn	Cudnn10.1
OpenCV	4.5.2

### 3.4 Training parameters

The training parameters of the training process used in the experiment are shown in **Table 3**.

**Table 3:** Training parameters.

Segmentation parameter	Value	Detection parameter	Value
Init_lr	0.007	Init_lr	0.01
Init_Epoch	0	Init_Epoch	0
Freeze_Epoch	50	Freeze_Epoch	50
Freeze_batch_size	8	Freeze_batch_size	8
UnFreeze_Epoch	330	UnFreeze_Epoch	620
Unfreeze_batch_size	4	Unfreeze_batch_size	4
Image Size	512*512	Image Size	640*640
Momentum	0.9	Momentum	0.937
Optimizer	sgd	Optimizer	sgd
backbone	MobileNetV2	Mixup_prob	0.5

### 3.5 Evaluation metrics

#### 3.5.1 Performance evaluation of segmentation

In this paper, several commonly used evaluation metrics for segmentation models are selected as segmentation performance evaluation metrics for the tea bud dataset, including pixel precision, mean pixel precision (mPA), intersection over Union (IoU), mean intersection over Union (mIoU), and mean recall (mRecall). Additionally, the number of parameters (Params) and frame rate (FPS) are also used as evaluation metrics for tea bud segmentation performance, taking into consideration the practical needs of tea bud segmentation.

The pixel precision metric represents the proportion of correctly predicted category pixels to the total number of pixels in the segmentation. The formula can be expressed as follows:


(7) $$\begin{array}{c} \;PA=\frac{\sum_{i=0}^{k}P_{ii}}{\sum_{i=0}^{k}\sum_{j=0}^{k}P_{ij}}\; \end{array}$$

The mean pixel precision represents the average value of the sum of the ratio of correctly predicted pixel points to the total pixel points for each category. The formula can be expressed as follows:


(8) $$\begin{array}{c} \;mPA=\frac{1}{k+1}\sum_{i=0}^{k}\frac{P_{ii}}{\sum_{j=0}^{k}P_{ij}}\; \end{array}$$

The Intersection over Union measures the overlap between the predicted and ground truth segmentation masks for each object category. It is defined as the ratio of the intersection between the predicted and ground truth masks to their union. The formula can be expressed as follows:


(9) $$\begin{array}{c} \;Iou=\frac{P_{ii}}{\sum_{j=0}^{N}P_{ij}+\sum_{j=0}^{N}P_{ji}-P_{ii}}\; \end{array}$$

The mean intersection over union represents the average of the intersection-over-union ratios between the predicted results and the ground truth labels for each category. It is a commonly used metric to evaluate the segmentation performance of models. The formula can be expressed as follows:


(10) $$\begin{array}{c} \;mIou=\frac{1}{k+1}\sum_{i=0}^{k}\frac{P_{ii}}{\sum_{j=0}^{k}P_{ij}+\sum_{j=0}^{k}P_{ji}-P_{ii}}\; \end{array}$$

The mean recall is the average ratio of the number of correctly classified pixels in each class to the total number of pixels in that class. The formula can be expressed as follows:


(11) $$\begin{array}{c} \;mRecall=\frac{1}{k+1}\sum_{i=0}^{k}\frac{P_{ii}}{P_{ii}+\sum_{j=0}^{k}P_{ji}}\; \end{array}$$

where *k* is the total number of categories, $P_{ij}$ represents the number of pixels that belong to class *i* but are predicted as class *j*, $P_{ii}$ represents the correct number predicted classes, and $P_{ji}$ is false positive or false negative.

#### 3.5.2 Performance evaluation of testing

To evaluate the detection model’s performance, this study uses precision(P), recall (R), mean average precision(mAP), and F1 score as evaluation metrics. Precision represents the proportion of true positive samples to all samples predicted as positive. The formula can be expressed as follows:


(12) $$\begin{array}{c} \;Presion=\frac{TP}{TP+FP}\; \end{array}$$

Recall represents the proportion of true positive samples to all positive samples in the dataset. The formula can be expressed as follows:


(13) $$\begin{array}{c} \;Recall=\frac{TP}{TP+FN}\; \end{array}$$

The formula shows that TP represents the number of predicted bounding boxes where the tea bud picking point is located, FP represents the number of predicted bounding boxes where the tea bud picking point is not located, and FN represents the number of missed bounding boxes where the tea bud picking point is located. Therefore, precision represents the proportion of correct predictions among all predicted outcomes, while recall represents the proportion of correct predictions among all true targets, where the values of both precision and recall are between 0 and 1.

The F1 score represents the weighted average of precision and completeness. The formula can be expressed as follows:


(14) $$\begin{array}{c} \;\text{F}1=\frac{2}{{\text{Recall}}^{-1}+{\text{Precision}}^{-1}}\;=2\;\cdot \;\frac{\text{Precision }\cdot \text{ Recall}}{\text{Precision }+\text{ Recall}}\; \end{array}$$

Precision reflects the ability of a model to correctly classify negative samples. A higher precision indicates a stronger ability of the model to distinguish negative samples. Recall, on the other hand, reflects the ability of the model to correctly identify positive samples. A higher recall indicates a stronger ability of the model to identify positive samples. F1 score is a combination of both precision and recall, where a higher F1 score indicates a more robust model.

The mean average precision represents the average value of the AP sought for all categories. The formula can be expressed as follows:


(15) $$\begin{array}{c} \;\text{mAP}=\frac{\sum_{\text{j}=1}^{\text{S}}\text{AP}\left(\text{j}\right)}{\text{S}}\; \end{array}$$

In the formula, S represents the number of all categories, and the numerator represents the sum of APs of all categories. Since this study only tested for tea bud picking points, the mAP can be calculated as mAP = AP.

### 3.6 MDY7-3PTB model

#### 3.6.1 *A* general overview *of the MDY7-3PTB* model

**Figure 7:**
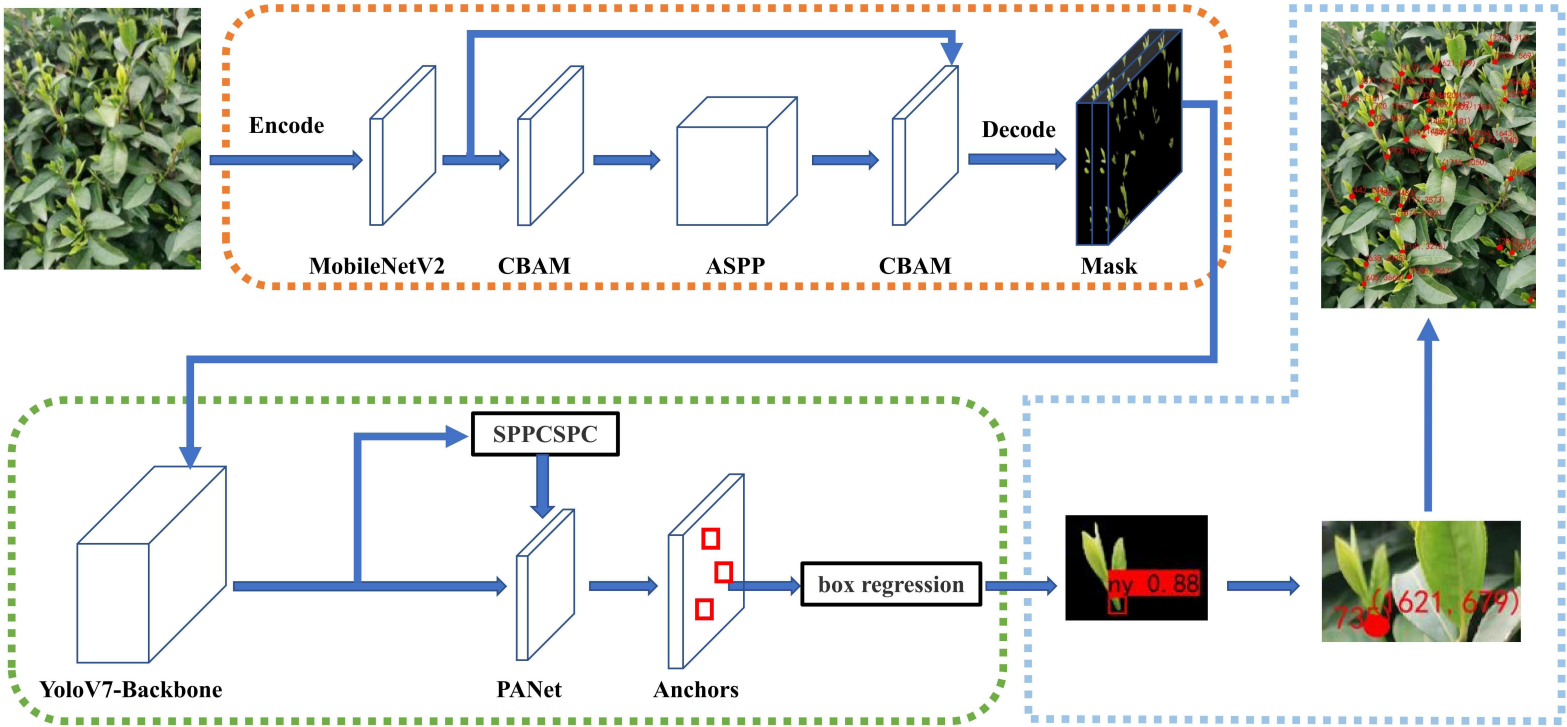
Overall overview of MDY7-3PTB model for tea bud segmentation, detection and localization.

The tea bud localization method proposed in this study, named MDY7-3PTB model, as shown in **Figure 7**. The MDY7-3PTB model combines the high-precision segmentation capability of DeepLabv3+ and the fast detection capability of YOLOv7. The process of locating tea bud picking points consists of three stages. In the first stage, which is the segmentation stage of tea buds, features are extracted from the input image using the MobileNetV2 backbone. Then, the feature maps are passed through the CBAM module to generate attention feature maps in both channel and spatial dimensions. These two feature maps are multiplied with the previous raw input feature map for adaptive feature recalibration, and then sent to the ASPP module. In the second stage, after the segmentation is completed, the detection of tea bud picking positions is performed. The segmented image is fed into the YOLOv7 backbone, which extracts features from the image, and then through the SPPCSPC module. This module addresses image distortion caused by image processing operations and the challenge of extracting repetitive features by convolutional neural networks. This approach is achieved by merging multiple MaxPool operations in a series of convolutions. Additionally, the FPN feature extractor combines high-level semantic information with low-level detail information to improve the detection of small targets. Finally, the fused features are input to box regression for boundary box correction, resulting in the predicted image of tea bud picking points. In the third stage, the predicted image is used to restore the background, and the two-dimensional coordinates are computed from the center of the rectangular boxes to obtain the tea bud picking points.

#### 3.6.2 Segmentation using MDY7-3PTB model

**Figure 8:**
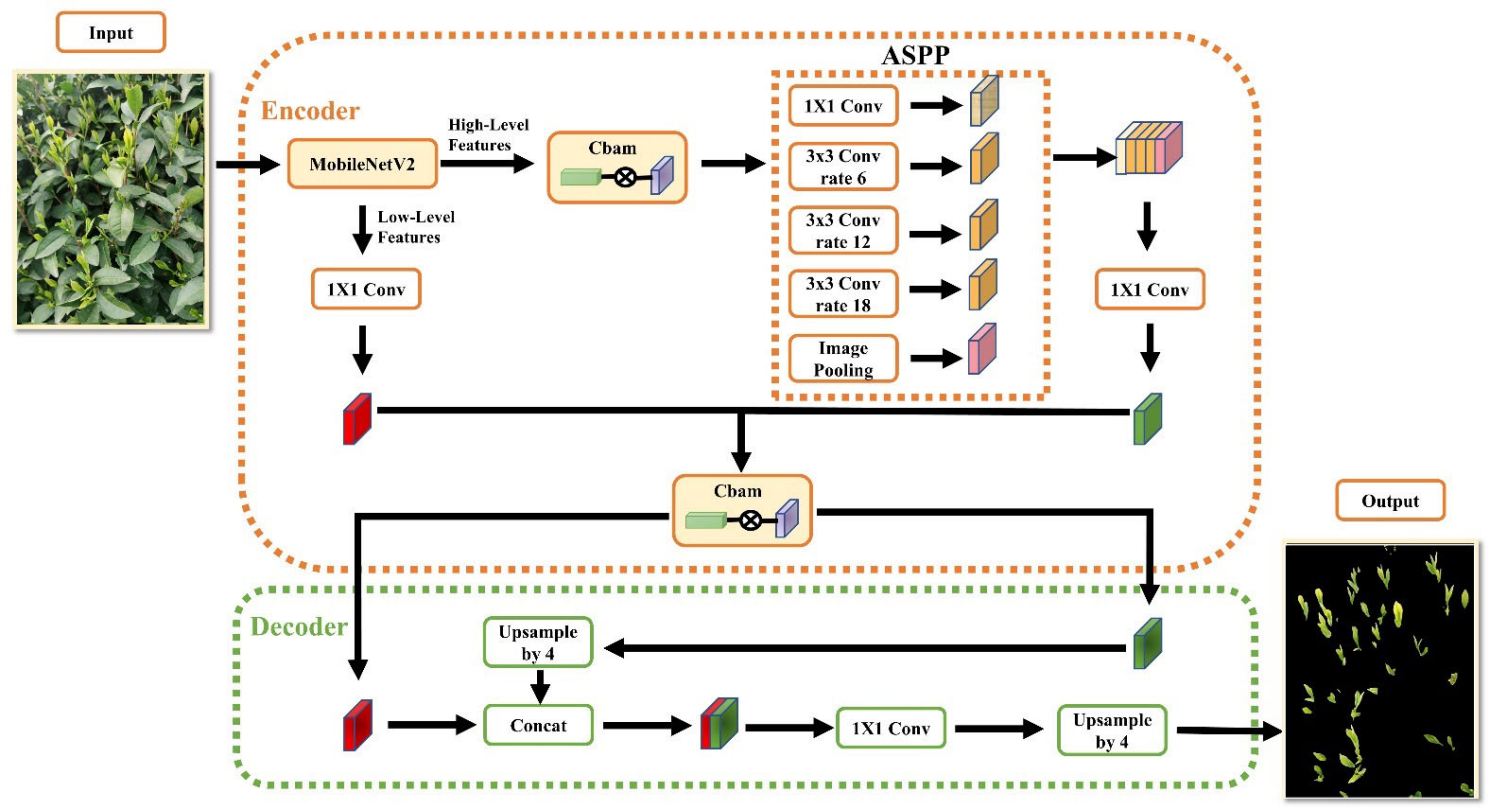
Improved DeeplabV3+ algorithmic network architecture.

DeepLabv3+ is currently the best-performing semantic segmentation model, however, it still has some limitations. For instance, the feature extraction network Xception has a large number of layers and parameters, leaving room for improvement in segmentation precision and operation speed. To enhance the segmentation effect of tea buds, this paper proposes several improvements to the DeepLabV3+ model. Firstly, the unsatisfactory Xception feature extraction network is replaced by the more lightweight MobileNetV2 network, significantly reducing the number of model parameters and improving calculation speed. Multiple fusion channels and the spatial attention mechanism CBAM are introduced before the feature extraction module and feature map input decoder to obtain better image features. Additionally, the weighted loss function is introduced to address the class imbalance problem of the dataset and improve the model’s segmentation precision for tea buds. The network structure of the improved DeepLabV3+ algorithm is illustrated in **Figure 8**.

#### 3.6.3 Detection using MDY7-3PTB model

To accurately detect segmented tea bud pictures, the MDY7-3PTB model utilizes the YOLOv7-Backbone as the detection backbone network. The labeled tea bud picking point dataset is fed into the YOLOv7-Backbone backbone network to extract picture features, which are then input into the SPPCSPC template to address issues such as image distortion caused by image processing operations and the problem of repeated features extracted from pictures by convolutional neural networks. After PANet feature fusion, the images are input to box regression for prediction box correction, resulting in the prediction of tea bud picking points. The detection backbone network consists of convolution, E-ELAN module, MPConv module, and SPPCSPC module. The E-ELAN module (Jiang et al., 2022), based on the original ELAN, modifies the computational blocks while retaining the transition layer structure of the original ELAN. It uses the concepts of expand, shuffle, and merge cardinality to achieve enhanced network learning without disrupting the original gradient path.The SPPCSPC module introduces multiple parallel MaxPool operations into a series of convolutions, which helps to prevent image distortion caused by image processing operations and the problem of extracting duplicate image features by convolutional neural network. In the MPConv module, the MaxPool operation expands the receptive field of the current feature layer and fuses it with the feature information after normal convolution processing, which improves the generalization of the network. The PANet module is a top-down and bottom-up bidirectional fusion backbone network with a “shortcut” between the bottom and top layers to shorten the path between layers. It also includes two modules, adaptive feature pooling and full connection fusion. The adaptive feature pooling can be used to aggregate features between different layers to ensure the integrity and diversity of features, and the full-connection fusion can achieve more accurate prediction results.

#### 3.6.4 Tea bud picking point positioning using the MDY7-3PTB model

**Figure 9:**
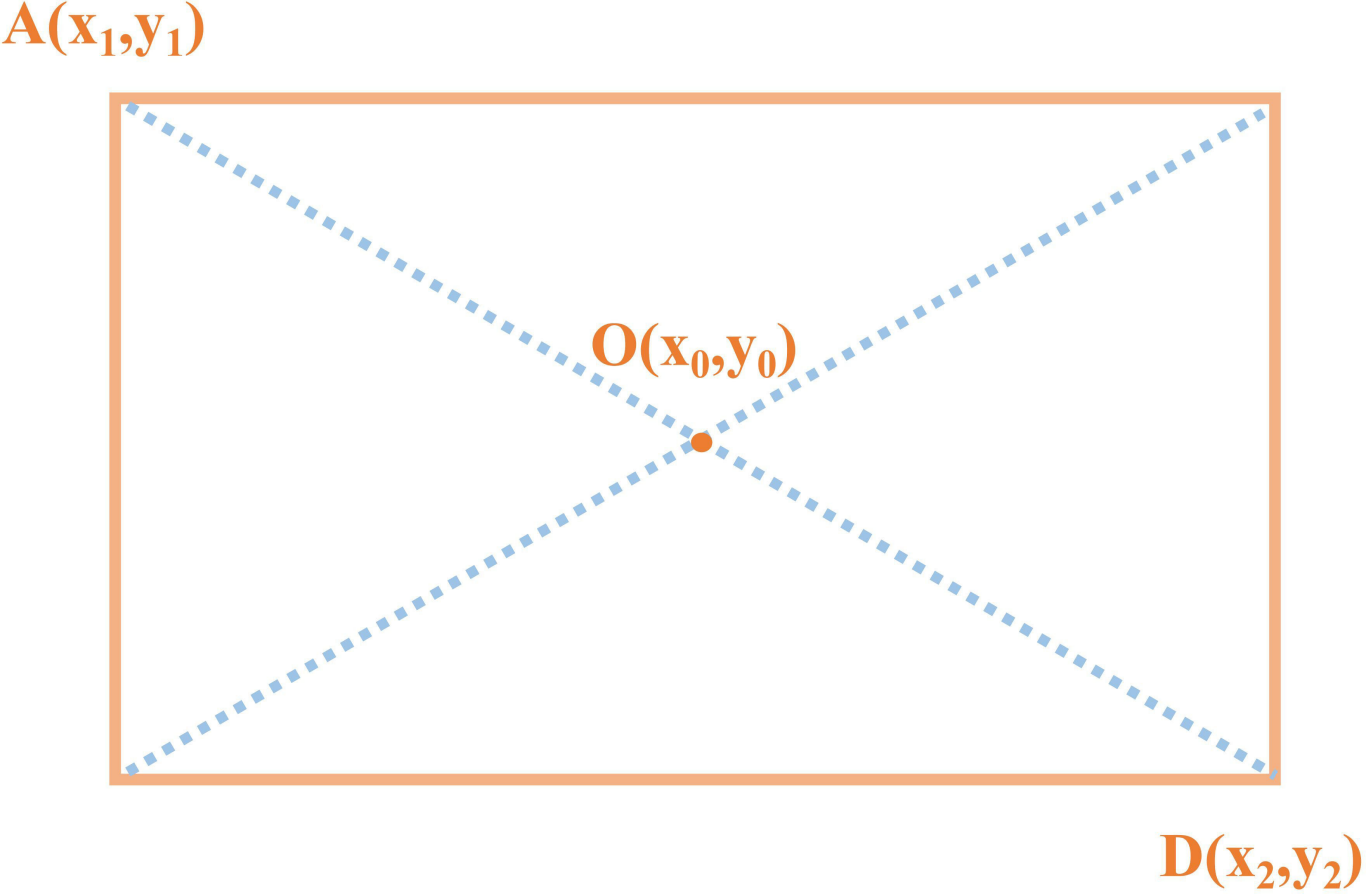
Calculation method for obtaining the center point of a rectangular box.


To obtain the two-dimensional coordinates of the tea buds, this study selects the geometric center of the prediction frame as the tea bud picking point coordinates, and applies the Box Regression module, which uses a 4-dimensional vector to represent the window, including the coordinates of the center point, width, and height. Finally, the window is adjusted through translation and scaling to gradually converge to the real value and obtain the exact coordinates of the tea bud picking point. Take the coordinates of A and D as shown in **Figure 9**. The formula for calculating the geometric center of the rectangle is as follows. $x_{0}$ and $y_{0}$ are the horizontal and vertical coordinates of the center point of the rectangular box. $x_{1}$ and $x_{2}$ is the horizontal coordinate of the vertex of the rectangle. $y_{1}$ and $y_{2}$ is the ordinate of the vertices of the rectangle.

(16) $$\begin{array}{c} \;x_{0}=\frac{x_{1}+x_{2}}{2}\; \end{array}$$

(17) $$\begin{array}{c} \;y_{0}=\frac{y_{1}+y_{2}}{2}\; \end{array}$$

## 4 Experimental results

### 4.1 Segmentation performance of the MDY7-3PTB model

As the segmentation results of tea shoot images can directly affect the subsequent identification and localization of picking points, segmentation precision is of primary concern. Additionally, the number of model parameters and segmentation speed, which are related to subsequent model deployment, should also be important factors to consider. To address these issues, this paper employs the Focal Loss function for data imbalance correction and proposes an improved DeeplabV3+ model, namely the MDY7-3PTB model, for segmentation. To further validate the segmentation performance of the MDY7-3PTB model, this study selected commonly used crop segmentation models such as PSPNet, Unet, and DeeplabV3+ for comparison. Additionally, different backbones were employed for the three comparative models, resulting in the establishment of six models: PSPNet_MobileNetV2, PSPNet_Resnet50, Unet_VGG, Unet_Resnet50, DeeplabV3+_MobileNetV2, and DeeplabV3+_Xception. In this study, 1000 images were selected for the training and testing of the segmentation model. The comparison results are presented in **Table 4**. The mIoU, mPA, and mRecall in **Table 4** represent the mean and standard deviation of the model’s results from 10 tests.

**Table 4:** Model performance table under multiple indicators.

Network Model	mIoU (%)	mPA (%)	mRecall (%)	Params (MB)	FPS
PSPNet_MobileNetV2	73.62 ±0.41	78.91 ±0.42	87.66 ±0.23	19.3	75.63
PSPNet_Resnet50	79.93 ±0.57	84.92 ±0.50	91.07 ±0.38	178.5	37.78
Unet_VGG	83.65 ±0.45	91.22 ±0.37	89.64 ±0.29	94.9	15.11
Unet_Resnet50	78.00 ±0.36	87.71 ±0.26	84.98 ±0.36	167.4	23.39
DeeplabV3+_MobileNetV2	80.43 ±0.40	88.89 ±0.40	87.26 ±0.36	22.4	63.12
DeeplabV3+_Xception	83.06 ±0.24	90.91 ±0.36	89.15 ±0.24	209.7	23.84
MDY7-3PTB	86.61 ±0.63	93.01 ±0.25	91.78 ±0.65	22.5	59.01

The results in **Table 4** demonstrate that the MDY7-3PTB model achieved a mean intersection over union of 86.61%, which outperforms other models such as DeeplabV3+_Xception, DeeplabV3+_MobileNetV2, Unet_Resnet50, Unet_VGG, PSPNet_Resnet50, and PSPNet_MobileNetV2, by 3.55%, 6.18%, 8.61%, 2.96%, 6.68%, and 12.99%, respectively. The MDY7-3PTB model also achieved a mean pixel precision of 93.01%, which increased by 2.10%, 4.12%, 5.30%, 1.79%, 8.09%, and 14.1%, respectively. Moreover, the MDY7-3PTB model showed a mean recall of 91.78%, which increased by 2.63%, 4.52%, 6.80%, 2.14%, 0.71%, and 4.12%, respectively. Additionally, the proposed method significantly reduced the number of model parameters and improved the FPS, resulting in faster and more accurate segmentation. To comprehensively evaluate the segmentation performance of the MDY7-3PTB model, the segmentation results of tea buds and background was compared and the results are presented in **Table 5**. The IoU, PA, and Recall in **Table 5** represent the mean and standard deviation of the model’s results from 10 tests.

**Table 5:** The results of different segmentation models on tea buds and backgrounds under multiple metrics.

Network Model	IoU (%)		PA (%)		Recall (%)	
	Tea Buds	Background	Tea Buds	Background	Tea Buds	Background
PSPNet_MobileNetV2	50.03 ±0.76	97.21 ±0.08	58.65 ±0.83	99.16 ±0.02	77.30 ±0.42	98.02 ±0.07
PSPNet_Resnet50	61.93 ±1.08	97.92 ±0.07	70.50 ±0.90	99.33 ±0.03	83.56 ±0.72	98.58 ±0.06
Unet_VGG	69.11 ±0.85	98.20 ±0.06	83.44 ±0.72	98.99 ±0.03	80.08 ±0.55	99.19 ±0.04
Unet_Resnet50	58.60 ±0.67	97.39 ±0.08	76.94 ±0.52	98.48 ±0.06	71.09 ±0.71	98.88 ±0.03
DeeplabV3+_MobileNetV2	63.09 ±0.75	97.78 ±0.06	79.15 ±0.77	98.76 ±0.03	75.67 ±0.67	98.98 ±0.05
DeeplabV3+_Xception	68.02 ±0.44	98.12 ±0.05	82.89 ±0.74	98.94 ±0.04	79.13 ±0.51	99.17 ±0.04
MDY7-3PTB	74.66 ±1.17	98.58 ±0.09	86.81 ±0.48	99.21 ±0.08	84.21 ±1.30	99.36 ±0.03

**Figure 10:**
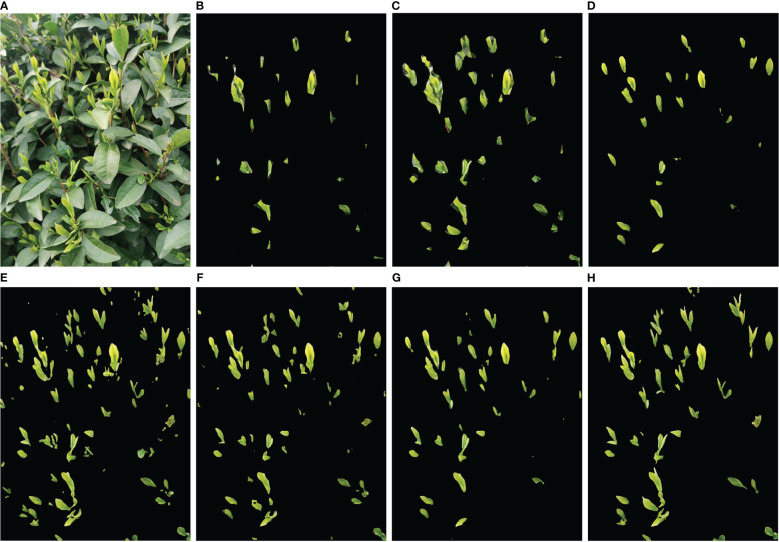
Examples of segmentation results for different models. **(A)** Original image; **(B)** PSPNet_MobileNetV2; **(C)** PSPNet_Resnet50; **(D)** Unet_VGG; **(E)** Unet_Resnet50; **(F)** DeeplabV3+_MobileNetV2; **(G)** DeeplabV3+_Xception; **(H)** MDY7-3PTB.

To further compare the performance of each method on tea bud segmentation, **Table 5** presents the results of intersection over union, pixel precision, and recall for both tea buds and background. It can be observed that the background has the highest values for intersection over union, pixel precision, and recall due to its large proportion in the image and small proportion of tea buds. Overall, MDY7-3PTB has higher values for intersection over union, pixel precision, and recall on both tea buds and background than the other comparison models, indicating that the proposed method is effective in improving the segmentation performance for each category. To provide a more clear and intuitive comparison of the tea bud prediction results of each model, this paper compiled the original image and segmentation maps of each network model. By adjusting parameters such as learning rate, threshold, and iteration, continuous training and optimization were performed to obtain the prediction results of each model. The comparison results are shown in **Figure 10**. **Figure 10** select a representative picture for display.

Based on the comparison chart above, it can be observed that MDY7-3PTB, DeeplabV3+Xception, and Unet_VGG models perform well in the actual tea bud segmentation effect, especially in terms of detailed contour aspects and small tea bud segmentation. Moreover, compared to DeeplabV3+Xception and Unet_VGG models for tea bud contour segmentation, MDY7-3PTB exhibits higher precision in tea bud segmentation, which is closer to the actual number of tea buds in the original image. However, other models have some segmentation defects. PSPNet MobileNetV2 and PSPNet Resnet50 models fail to segment many tea buds accurately, and some of the segmented tea bud backgrounds is not completely removed, leading to low tea bud segmentation precision. Unet_Resnet50 and DeeplabV3+_MobileNetV2 models suffer from segmentation errors, where some tea leaves are erroneously segmented as tea buds, and the background of a few tea buds is not entirely removed.

Based on the comparison of the result data and image analysis above, it can be concluded that the proposed MDY7-3PTB model has significantly improved the segmentation precision, and the model is also lightweight and more suitable for actual tea bud segmentation needs.

### 4.2 Detection performance of the MDY7-3PTB model

After the dataset in this study is segmented and then input the segmented dataset into YOLOv3, YOLOv4, YOLOv5s, YOLOxm, and YOLOv7 detection models to create the corresponding MDY3-3PTB, MDY4-3PTB, MDY5s-3PTB, MDYxm-3PTB, and MDY7-3PTB models. The YOLOv7 module represents direct detection of the original image. The MDY3-3PTB module combines the high-precision segmentation capability of improved DeeplabV3+ module and the rapid detection of YOLOv3, the MDY4-3PTB module combines the high-precision segmentation capability of improved DeeplabV3+ module and the rapid detection of YOLOv4, and the MDY5s-3PTB module combines the high-precision segmentation capability of improved DeeplabV3+ module and the rapid detection of YOLOv5s, and so on. This paper compares their evaluation metrics such as precision, recall, F1 score, and mAP_(@0.5), as shown in **Table 6**. The “mAP@0.5” in **Table 6** refers to the mAP value calculated at a confidence threshold of 0.5.

**Table 6:** Comparison of segmentation performance under multiple metrics.

Network Model	P	R	F1	${\text{mAP}}_{@0.5}$
YOLOv7	85.64	71.50	77.93	79.82
MDY3-3PTB	74.22	68.19	71.08	63.48
MDY4-3PTB	70.12	53.26	60.54	53.75
MDY5s-3PTB	89.42	61.93	73.18	74.86
MDYxm-3PTB	95.51	91.15	93.28	91.62
MDY7-3PTB	97.27	89.41	93.17	93.52

After comparing the results presented in **Table 6**, it is evident that the overall performance of the MDY7-3PTB model proposed in this paper is superior to other models. Specifically, the precision of MDY7-3PTB is 97.27%, which is higher than YOLOv7, MDY3-3PTB, MDY4-3PTB, MDY5s-3PTB, MDYxm-3PTB, and MDY7-3PTB models by 11.63%, 23.05%, 27.15%, 7.85%, and 1.76% respectively. The recall of MDY7-3PTB is 89.41%, which represents an improvement of 17.91%, 21.22%, 36.15%, 27.48%, and -1.74% respectively. In terms of F1 score, MDY7-3PTB achieves a value of 93.17%, which is higher than YOLOv7, MDY3-3PTB, MDY4-3PTB, MDY5s-3PTB, and MDYxm-3PTB by 15.24%, 22.09%, 32.63%, 19.99%, and -0.11% respectively. Moreover, in terms of the mean average precision, MDY7-3PTB is 93.52%, which represents an improvement of 13.7%, 30.04%, 39.77%, 18.66%, and 1.9% respectively.

**Figure 11:**
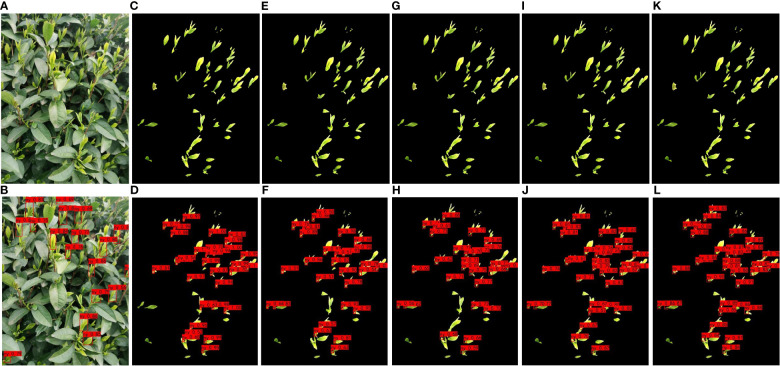
Examples of detection results of different models for segmented datasets; **(A, B)** YOLOv7; **(C, D)** MDY3-3PTB; **(E, F)** MDY4-3PTB; **(G, H)** MDY5s-3PTB; **(I, J)** MDYxm-3PTB; **(K, L)** MDY7-3PTB.

To facilitate a clearer and more intuitive comparison of the tea bud picking point prediction results from each model, this paper presents a collation of the prediction results from each network model. By adjusting parameters such as learning rate, threshold, and iteration, continuous training and optimization were conducted, and ultimately, the prediction results of each model were obtained. The comparison of their effects is presented in **Figure 11**. **Figure 11** select representative pictures for display.

Based on the comparison chart above, it can be concluded that the MDY7-3PTB model has the best performance in actual tea bud picking point detection. The MDY7-3PTB model has no missed or erroneous detections and high confidence, while the other models have their own limitations. The MDYxm-3PTB model has a detection performance similar to that of MDY7-3PTB, but with a few missed detections and a lower confidence level. The MDY5s-3PTB, MDY4-3PTB, and MDY3-3PTB models have worse performance in all aspects, with many missed detections and tea bud picking points detected with relatively low confidence. The YOLOv7 model, which directly detects tea buds from the original images, is much less effective than MDY7-3PTB and other models, and shows significant missed detection, which can be attributed to the similar color of tea buds and tea leaves in complex environments. Therefore, it is better to remove the background and perform data enhancement before detection. Furthermore, from the predicted images of each model, it is observed that the picking points of tea buds captured from side views are easier to detect, while the picking points of tea buds captured from top views are more challenging to detect, which requires further investigation in future studies.

**Figure 12:**
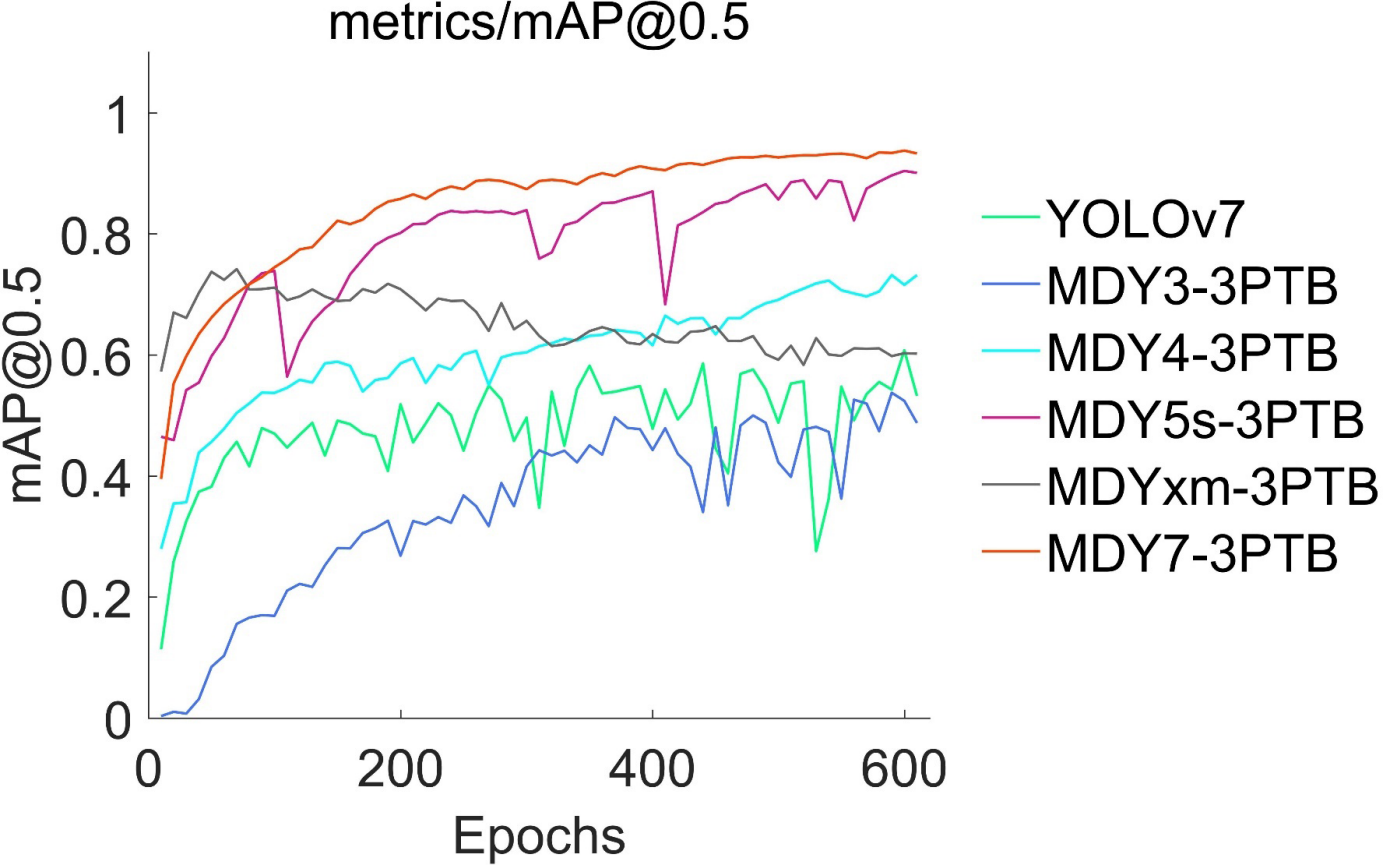
Accuracy variation of six object detectors.

**Figure 13:**
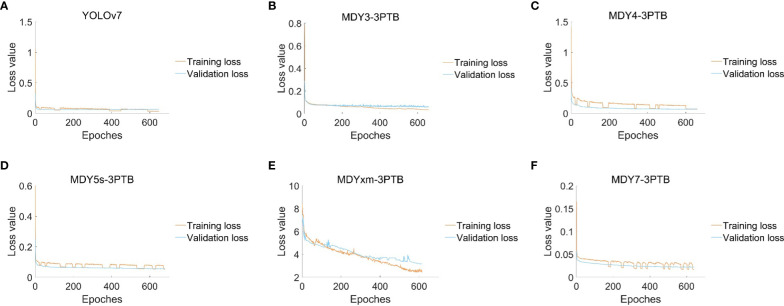
**(A)** The loss changes of the YOLOv7 model; **(B)** The loss changes of the MDY3-3PTB model; **(C)** The loss changes of the MDY4-3PTB model; **(D)** The loss changes of the MDY5s-3PTB model; **(E)** The loss changes of the MDYxm-3PTB model; **(F)** The loss changes of the MDY7-3PTB model.

This paper presents experimental data on the training conditions of a model for tea bud picking point detection, using the change of mean average precision (mAP) and loss function as indicators. A scatter diagram is used to visualize the results. The proposed MDY7-3PTB model is compared to five mainstream YOLO detection models, and its advantages in detection precision are demonstrated. The other models, including MDY3-3PTB, MDY4-3PTB, MDY5s-3PTB, MDYxm-3PTB, and YOLOv7, have various shortcomings in detection. As the iteration times increase, the mAP of MDY7-3PTB significantly outperforms that of other models. Compared to the original YOLOv7, the MDY7-3PTB model converges much faster due to the early removal of background interference and the addition of the CBAM attention mechanism, which enhances the feature distribution weights of objects in both the spatial and channel dimensions and eliminates the interference of irrelevant features. Although MDYxm-3PTB is second only to MDY7-3PTB in all aspects of metrics, it has slower convergence and larger early fluctuations. These findings are shown in **Figures 12**, **13**.

In summary, based on the result data and image comparisons presented above, it is evident that the MDY7-3PTB model proposed in this paper has significantly improved detection precision. This provides a solid theoretical foundation for future developments in tea bud picking.

### 4.3 Positioning performance of the MDY7-3PTB model

**Figure 14:**
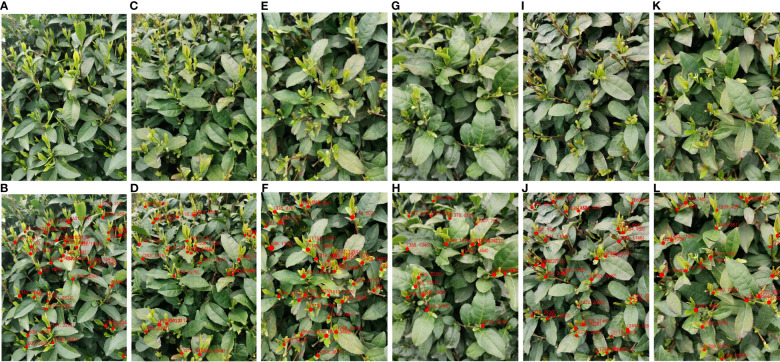
Example of results of MDY7-3PTB model for locating picking points of tea buds in complex environments; **(A, C, E, G, I, K)** for original images; **(B, D, F, H, J, L)** for tea bud picking points identified.

To provide a theoretical basis for the tea-picking robot, this paper uses MDY7-3PTB to segment and detect the tea bud picking points, and outputs the coordinates of the picking points by taking the geometric center of the rectangular frame and restoring their background, achieving accurate localization of the tea bud picking points. The two-dimensional coordinate positioning is shown in **Figure 14**, where the set in parentheses indicates the position coordinates of the tea bud picking point relative to the original image. Excluding the tea buds that were not segmented due to focusing issues, the number of tea bud picking points marked after segmentation was 167, with 161 correct positioning and a positioning precision of 96.41%. This greatly improved the precision compared to direct detection and subsequent positioning, thereby enhancing the precision of subsequent tea bud picking.

## 5 Discussion

As is well known, some researchers have used the YOLO series detection algorithm for detecting and classifying tea buds, as well as other crops such as citrus (Chen et al., 2022), apple (Sun et al., 2022), grape (Yang et al., 2022), lychee (Xie et al., 2022), and camellia oleifera fruit (Wu et al., 2022). These crops have significantly different colors from their backgrounds, making it easy to accurately locate their positions. In addition, these crops are round in shape and different from the surrounding background, such as leaves, making it possible to directly use the detection algorithm for object detection with high accuracy. However, tea buds have similar colors and shapes to their background, so using the detection algorithm directly for detection may result in problems such as missed detection, false detection, low detection speed, and low accuracy.

Therefore, to eliminate background interference and improve the accuracy of tea bud localization, this study first performs segmentation on the tea buds, separating them from the background. Then, object detection is applied to the segmented tea buds to identify the picking points, followed by the final localization of the tea buds. The addition of an attention mechanism module is an effective way to enhance model performance (Chen et al., 2022; Khan et al., 2022). The proposed improved DeepLabV3+ model based on attention mechanism can effectively accomplish the segmentation task. Furthermore, compared to directly performing tea bud object detection (Li et al., 2023) or segmentation alone, the proposed approach of combining object segmentation and detection in this study achieves better results (Guo et al., 2023; Meng et al., 2023). However, there are still a very few tea buds whose coordinates have not been correctly identified, which can be attributed to the following reasons summarized as follows: Firstly, the tea buds in the images are excessively blurred, leading to segmentation or detection failures. Secondly, several tea buds overlap with each other, causing incomplete segmentation or detection. Thirdly, some tea buds only partially appear in the images, resulting in recognition errors.

Currently, Zhang et al. (2021) use a binocular depth camera to capture images of fruit in the field and design a fruit spatial positioning system. Yan et al. (2022) use the MR3P-TS model to locate tea picking points and provide cutting angle recommendations. Xu et al. (2022) proposed a combined multi-point picking scheme and selectively designed the size of the tip of the bud picker. Chen et al. (2022) apply robot technology and deep learning to develop a computer vision system for intelligent tea picking, providing theoretical support for the intelligent picking of tea buds. The above research indicates that achieving automated picking of tea buds requires particular emphasis on the localization of picking points. The novel method proposed in this study for tea bud picking point localization can effectively accomplish this task, providing a theoretical foundation for the development of tea-picking robots.

## 6 Conclusion

The tea plantation background is complex, and manual picking requires a lot of manpower and resources. In this paper, we propose a MDY7-3PTB model based on the high-precision segmentation ability of DeepLabv3+ and the fast detection ability of YOLOv7, which is mainly used for tea leaf detection and localization in tea plantations. The model consists of three stages. The first stage uses the Focal Loss function to correct the class imbalance in the original dataset, and then uses the CBAM attention mechanism and lightweight DeepLabv3+ network to segment the original tea leaf dataset. The second stage uses YOLOv7 to detect the tea picking points after segmentation. The third stage uses the method of taking the center of the rectangular box to accurately locate the two-dimensional coordinates of the tea picking points. In testing, the proposed MDY7-3PTB model was compared with other object segmentation or detection models. The model achieved an average intersection over union (IoU) of 86.61%, an average pixel accuracy of 93.01%, and an average recall of 91.78% on the tea shoot segmentation dataset. In addition, for tea plucking point recognition and localization, the model showed significant improvements compared to existing mainstream detection models, with a mean average precision of 93.52%, a weighted average of precision and recall of 93.17%, a precision of 97.27%, and a recall of 89.41%. These results provide a strong theoretical foundation for the future of tea plucking, demonstrating significant advancements in all aspects of the detection process. In future research, the goal is to develop a system for annotating the three-dimensional coordinates of tea picking points using a binocular depth camera, which can be combined with mechanical structures to provide further theoretical basis for intelligent tea picking and achieve fine tea picking. Additionally, Future research will focus on improving the YOLOv7 model by adding attention mechanism modules, reducing the number of parameters, and increasing the detection speed.

## Data availability statement

The raw data supporting the conclusions of this article will be made available by the authors, without undue reservation.

## Author contributions

FZ: Methodology, Analysis, Suggestion, Writing-review. SX: Conceptualization, Investigation, Writing-original draft, Writing-review and editing. CD: provided the tea buds data. LY: Methodology, Analysis, Writing-review. YX and ZZ: Picture Drawing and Artistic Design. HS and FC: Validation, Resources, Supervision, Project administration, Writing-review and editing, Funding acquisition. All authors contributed to the article and approved the submitted version.
